# Supplementary material for: Preparation of Electrospun Small Intestinal Submucosa/Poly(caprolactone-co-Lactide-co-glycolide) Nanofiber Sheet as a Potential Drug Carrier
Source: Pharmaceutics. 2021 Feb 11;13(2):253. doi: 10.3390/pharmaceutics13020253 (PMC7917610; doi:10.3390/pharmaceutics13020253)
Supplement: Supplementary file 1 [file pharmaceutics-13-00253-s001.pdf]

pharmaceutics

MDPI

Supplementary Materials: Preparation of Electrospun Small Intestinal Submucosa/poly(caprolactone-Co-Lactide-Co-glycolide) Nanofiber Sheet as a Potential Drug Carrier

Nguyen Thi Thu Thao †, Surha Lee †, Gi Ru Shin †, Youngji Kang, Sangdun Choi, Moon Suk Kim *

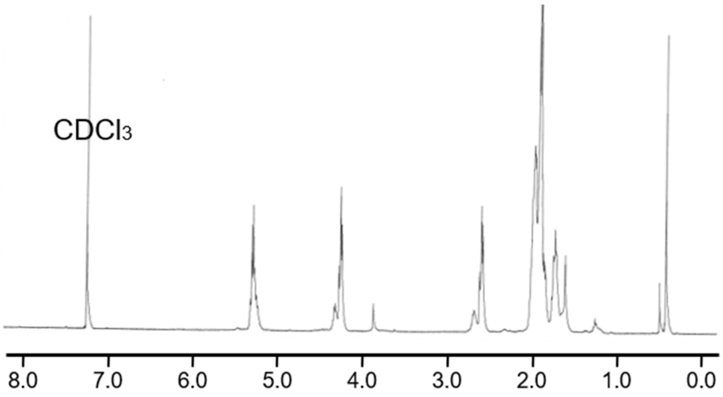

Figure S1. ^{1}H-NMR of PCLA in CH_2Cl_2 soluble part of S/P-NS.

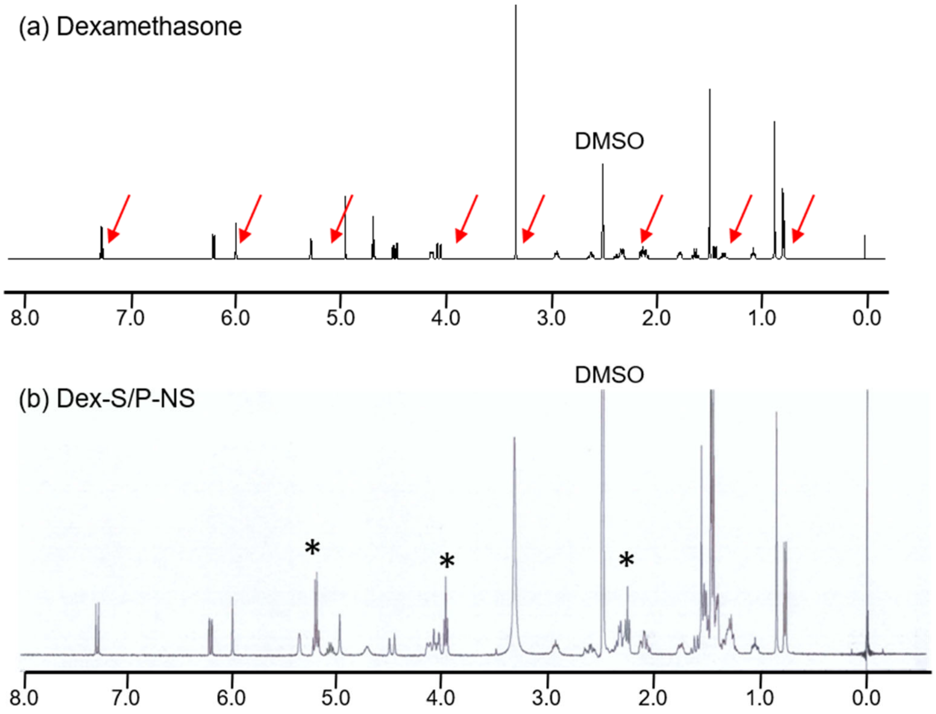

Figure S2. ^{1}H-NMR of (a) dexamethasone and (b) DMSO solubilized part of Dex-S/P-NS (* indicated the PCLA assignable peak).

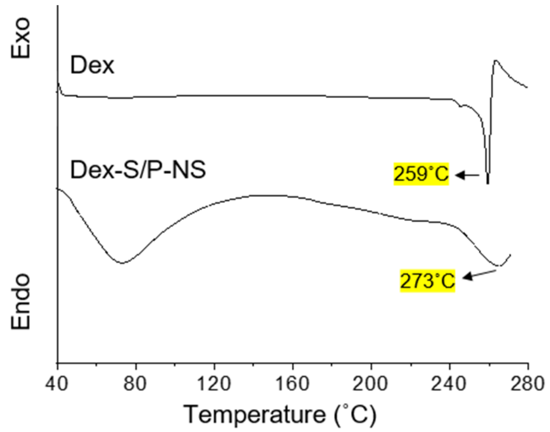

Figure S3. DSC of (a) dexamethasone and (b) Dex-S/P-NS (yellow highlight indicated the melting temperature assignable to Dex).

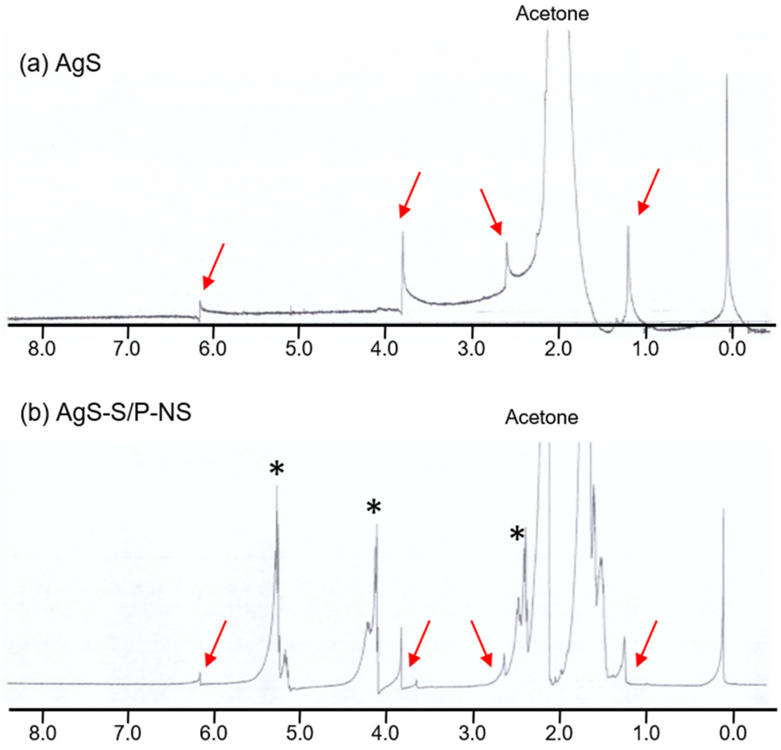

Figure S4. ^{1}H-NMR of (a) AgS and (b) acetone solubilized part of AgS-S/P-NS (* indicated the PCLA assignable peak and arrows indicated AgS).

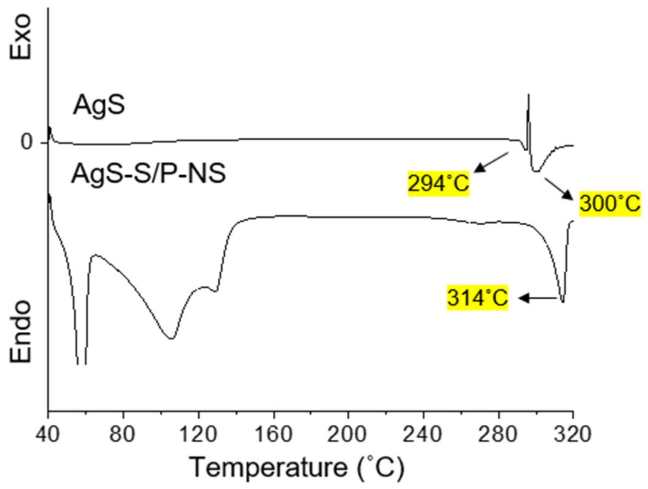

Figure S5. DSC of (a) AgS and (b) AgS-S/P-NS (yellow highlight indicated the melting temperature assignable to AgS).

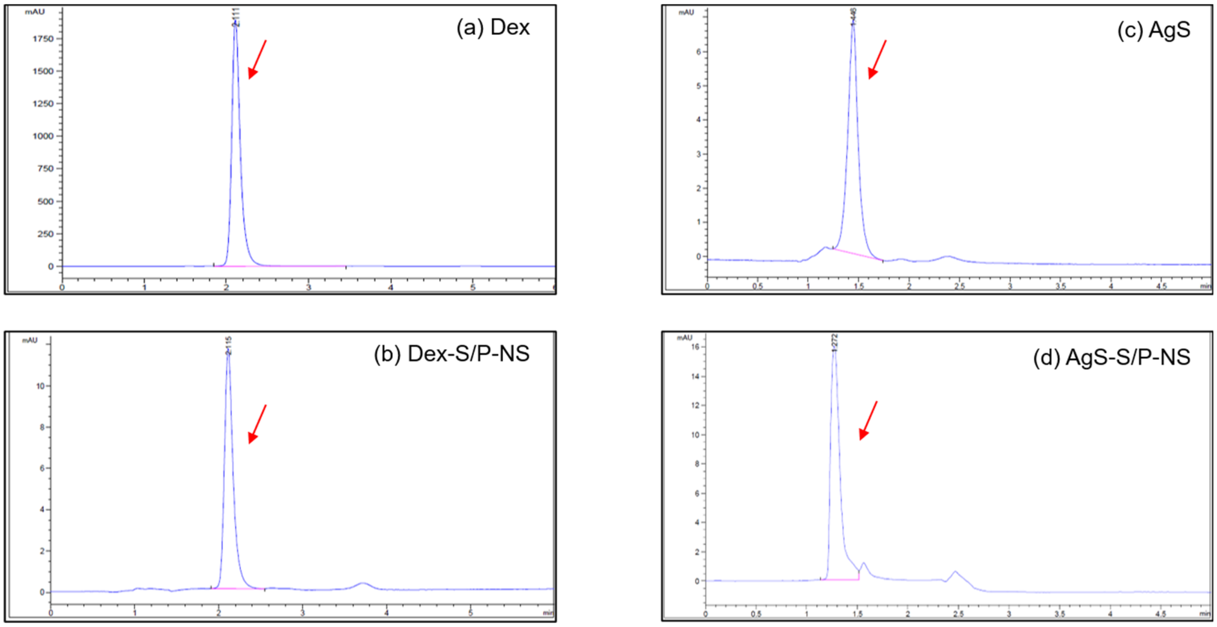

Figure S6. HPLC graph of (a) native Dex and AgS alone and (b) the released Dex from Dex-S/P-NS and AgS from AgS-S/P-NS (arrow indicated the peaks of native Dex or AgS and the released Dex or AgS from corresponding S/P-NS).

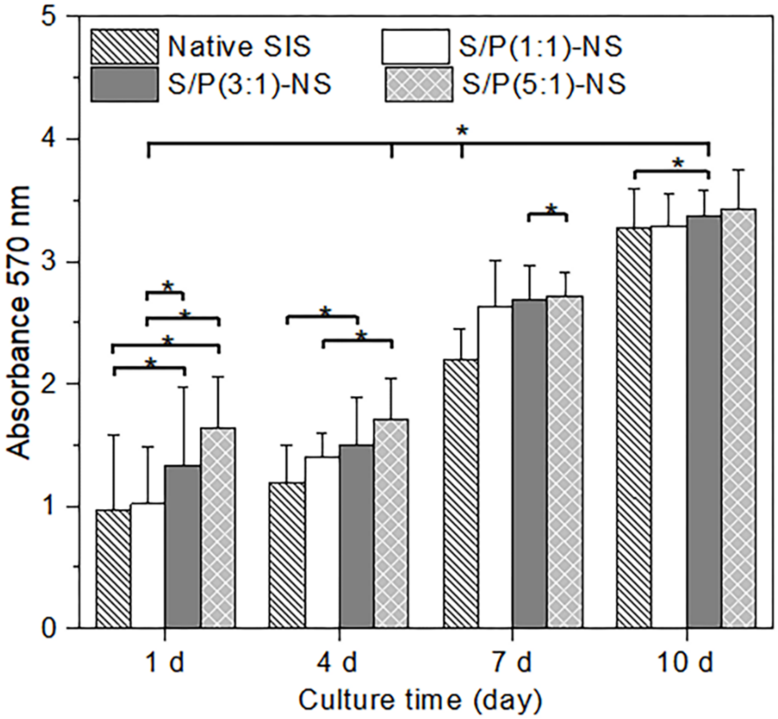

Figure S7. Proliferation of NIH 3T3 cells on the native SIS sheet, native SIS sheet, S/P(5:1)-NS, S/P(3:1)-NS and S/P(1:1)-NS for 10 days (*$p < 0.05$).
